# Profoundly Uncontrolled Diabetes Mellitus and Social Disadvantage Among Hospitalized Patients with Mucormycosis in Central California

**DOI:** 10.3390/jof11110765

**Published:** 2025-10-24

**Authors:** Almira Opardija, Krishna Ragavachari Suresh, Pavel Diaz, Yueqi Yan, Geetha Sivasubramanian

**Affiliations:** 1 Division of Infectious Diseases, Department of Medicine, University of California San Francisco, Fresno, CA 93701, USA; 2 Department of Internal Medicine, University of South Dakota, Vermillion, SD 57069, USA; 3 School of Engineering, University of California, Merced, CA 95343, USA; 4 HSRI Biostatistics and Data Support Core, University of California, Merced, CA 95343, USA; yyan105@ucmerced.edu

**Keywords:** mucormycosis, social deprivation, uncontrolled diabetes mellitus

## Abstract

Mucormycosis (MCM) is an opportunistic fungal infection in immunocompromised hosts, most commonly associated with poorly controlled diabetes mellitus (DM). We conducted a retrospective review of 45 MCM cases diagnosed between 2010 and 2023 at a referral center in Central California, a region with high DM prevalence and significant healthcare disparities. Clinical features, histopathology, microbiology, treatment, and outcomes were analyzed. Ninety-six percent of patients had DM, and 69% had no other predisposing condition. Glycemic control was markedly poor: 36% had HbA1c > 10%, and 61% had HbA1c > 8%. Diabetic ketoacidosis (DKA) was present in 19% of patients and associated with 100% mortality. Rhino-orbito-cerebral mucormycosis (ROCM) accounted for 60% of cases and carried a 70% mortality rate. Angioinvasion, confirmed in 62% of biopsied cases, significantly increased mortality (69% vs. 28%, *p* = 0.015). In-hospital mortality remained high at 58%, consistent with outcomes reported in other high-burden settings. Over 60% of patients identified as Hispanic. ZIP code–based analyses revealed that 75% of individuals lived in neighborhoods with Healthy Places Index (HPI) scores below the 25th percentile, and 64% resided in areas with a Social Deprivation Index (SDI) of 85 or higher, indicating entrenched structural disadvantage. Our findings highlight that MCM in Central California disproportionately affects individuals with uncontrolled DM living in socially deprived areas. These data underscore the need for early diagnosis, targeted antifungal therapy, and upstream public health interventions addressing diabetes management and healthcare access.

## 1. Introduction

Mucormycosis (MCM), a disease caused by filamentous fungi belonging to the order Mucorales, represents a serious health concern, particularly among individuals with weakened immune defenses [1]. These organisms are ubiquitous in the environment and thrive on decaying organic matter. Infection typically begins with the inhalation of fungal spores into the paranasal sinuses or lower respiratory tract [2]. The organism then causes a locally aggressive, angioinvasive infection that may spread contiguously to adjacent structures or disseminate hematogenously to distant sites, including the central nervous system [3]. The progression and outcome of mucormycosis depend on multiple factors, including the timeliness of diagnosis, access to appropriate medical and surgical care, the patient’s underlying immune status, and the inherently aggressive nature of the pathogen. Clinical outcomes range from successful management with antifungal therapy and debridement to rapid progression and death [4,5].

Presentations include sinopulmonary involvement, rhino-orbito-cerebral mucormycosis (ROCM), cutaneous lesions, gastrointestinal disease, and disseminated infections [6]. Invasive forms are associated with high mortality rates, typically between 30–50% and exceeding 90% in disseminated cases. The angioinvasive and thrombosis-inducing nature of MCM, particularly in the context of uncontrolled diabetes mellitus (DM) or immunosuppression, contributes to extensive tissue destruction [7]. Timely recognition and intervention are critical for improving survival; however, management often necessitates extensive surgical debridement, which may result in significant tissue loss and potential disfigurement [8].

Well-established risk factors include poorly controlled DM, iron overload, acidic physiological states (e.g., diabetic ketoacidosis (DKA)), hematologic malignancies, organ transplantation, neutropenia, corticosteroid exposure, and protein-energy malnutrition [9]. Failure to address these underlying conditions can foster rapid angioinvasive fungal growth, often going undetected until advanced stages requiring radical interventions.

The rising global burden of diabetes further amplifies the threat of MCM. According to the 2021 Global Burden of Disease (GBD) study, there has been a marked increase in type 2 DM among non-elderly individuals between 1990 and 2021 [10]. In low-resource settings such as parts of Asia, uncontrolled DM is the predominant risk factor for MCM, while in high-income countries, it is more often associated with hematologic malignancies and transplantation [11]. In the United States, the rural Central Valley of California presents a uniquely challenging landscape. As a major agricultural hub, the region faces pronounced socioeconomic and healthcare disparities, including limited access to specialty care, high rates of poverty, and low health literacy, paralleling trends observed in other resource-limited environments [12]. Additionally, from 2013 to 2017, the age-adjusted prevalence of DM among adults in California rose from 8.7% to 10.7%, with Central California counties consistently reporting some of the highest rates in the state [13].

These challenges contribute to widespread delays in diagnosis and suboptimal chronic disease management. The convergence of poor glycemic control and healthcare disparities creates a permissive environment for opportunistic infections such as mucormycosis. As the region grapples with an expanding diabetes epidemic, driven in part by limited healthcare access, poverty, and other structural barriers to chronic disease management, this deadly fungal infection warrants greater scrutiny. This study aims to characterize the clinical presentation, risk factors, and outcomes of mucormycosis in adults treated at a major referral center in Central California, highlighting an underrecognized but severe opportunistic infection in this region.

## 2. Materials and Methods

We conducted a retrospective review of all cases of mucormycosis (MCM) diagnosed at a major academic referral hospital in Fresno, California, between 1 January 2010, and 31 December 2023. Cases were identified using International Classification of Diseases, Ninth and Tenth Revision (ICD-9 and ICD-10) codes. Medical records were reviewed to confirm eligibility. Patients were included if they were ≥18 years of age and had a diagnosis of MCM confirmed by either a positive fungal culture and/or histopathologic evidence of invasive Mucorales infection.

Fungal cultures were reviewed from relevant clinical specimens, including sinus, pulmonary, cutaneous, muscular, and gastrointestinal sites. All isolates were identified in-house at the Community Regional Medical Center (CRMC) Microbiology Laboratory in Fresno, California, using standard phenotypic methods. Advanced diagnostic techniques such as MALDI-TOF or fungal PCR/sequencing were not employed. Histopathologic diagnosis required the presence of broad, ribbon-like, sparsely septated hyphae demonstrating tissue invasion, necrosis, or angioinvasion, consistent with Mucorales morphology. Cases showing only fungal elements without tissue damage were excluded, in accordance with the EORTC/MSGERC definitions for proven invasive fungal disease [14]. Cases showing only fungal elements without tissue damage were excluded, in accordance with the EORTC/MSGERC definitions for proven invasive fungal disease [14].

Demographic data, comorbidities, clinical presentation, microbiological and histopathologic findings, laboratory results, antifungal therapy, surgical interventions, and in-hospital mortality were extracted. The period of outcome assessment was limited to the index hospitalization; no post-discharge mortality or recurrence data were available. The EQUAL Score for Mucormycosis (European Confederation of Medical Mycology, 2018) was applied to assess diagnostic and therapeutic quality [15]. This structured tool allocates weighted points across diagnostic, treatment, and follow-up domains, with a total achievable score ranging from 25 to 32 points, depending on whether culture and/or histopathologic confirmation were obtained [15].

As individual-level data on social determinants of health (SDOH), such as education, occupation, transportation access, and health literacy, were unavailable in this retrospective review, we used ZIP Code Tabulation Areas (ZCTAs) to estimate community-level deprivation. We extracted Social Deprivation Index (SDI) scores, developed by the Robert Graham Center, and California Healthy Places Index (HPI) scores, from the Public Health Alliance of Southern California. SDI is a composite metric (0–100; higher = more deprivation) based on census-derived socioeconomic indicators. HPI is a percentile-based tool (0–100; higher = greater community health opportunity) [16,17]. Both have been widely used as proxies for health equity, social disadvantage, and access to care [18]. Due to small sample size and geographic heterogeneity, these data were summarized descriptively but not included in the outcomes analysis.

The primary outcomes were in-hospital mortality, number of surgical debridements, and duration of antifungal therapy. Amphotericin B–associated toxicities were also recorded. This study was approved by the institutional review board. Descriptive statistics were used to summarize baseline characteristics. Categorical variables were analyzed using the χ^2^ test or Fisher’s exact test, and continuous variables were compared using the Wilcoxon rank-sum test. A two-tailed *p*-value < 0.05 was considered statistically significant.

## 3. Results

**Figure 1:**
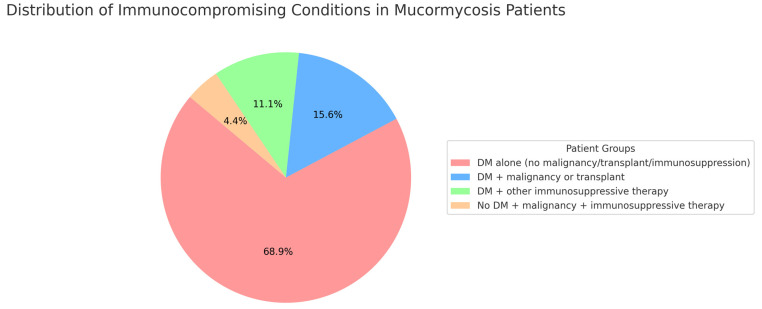
Distribution of Underlying Conditions in Patients with Mucormycosis. This figure highlights the predominance of diabetes mellitus as the primary risk factor in this Central California cohort.

A total of 45 patients with MCM were admitted between 2010 and 2023. The median age was 53 years (interquartile range [IQR], 18–81); 64% were male, and 62% identified as Hispanic (Table 1). Diabetes mellitus (DM) was the predominant underlying condition in this cohort (Figure 1). Of the 45 patients with MCM, 43 (96%) had underlying DM. Among these, 31 patients (69%) had DM alone without any concurrent malignancy, transplant, or use of immunosuppressive therapy. The remaining 12 diabetic patients (27%) had an additional immunocompromising condition, including malignancy or transplant (n = 7) and/or immunosuppressive therapy for other conditions (n = 5). Only two patients (4%) did not have diabetes; both had malignancy and were receiving immunosuppressive therapy, representing the only non-diabetic immunocompromised individuals in the cohort.

This table summarizes baseline demographic characteristics, diabetes status, comorbid immunocompromising conditions, and laboratory parameters related to glycemic and iron metabolism. Data are presented as counts and percentages or means with standard deviations where applicable.

**Figure 2:**
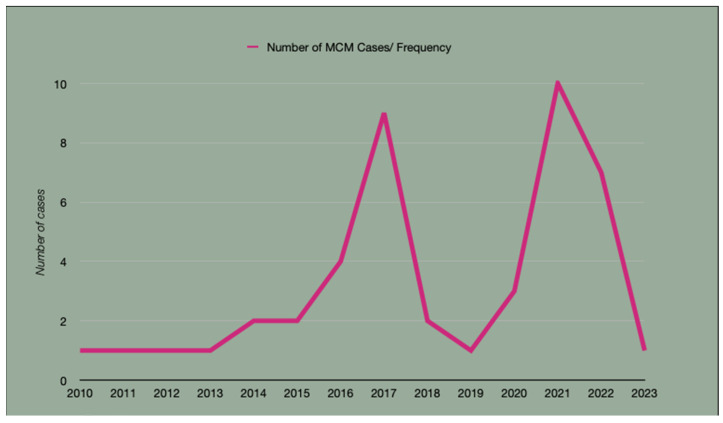
Annual number of mucormycosis cases diagnosed at a Central California referral center from 2010 to 2023. Notable spikes occurred in 2017 and 2021–2022, potentially correlating with regional flooding events and the COVID-19 pandemic.

A notable increase in cases was observed in 2017 (n = 9) and again in 2021–2022 (n = 17). Since the onset of the SARS-CoV-2 pandemic, 21 cases were diagnosed (Figure 2); however, only 8 had documented COVID-19 infection prior to MCM onset, and half of those required hospitalization for COVID-related complications. Data on corticosteroid use were not consistently recorded and could not be analyzed.

ROCM was the most frequent presentation (60%), followed by pulmonary (22%), muscular (9%), cutaneous (7%), and gastrointestinal involvement (2%).

Among patients with DM, 19% presented with DKA. Hemoglobin A1c values were available for 41 patients: 24 (58%) had levels > 7.5%, and 15 (36%) had levels > 10%. The mean HbA1c was 9.5%, while the median was 7.8% (IQR, 6.7–12.6), reflecting a right-skewed distribution with several patients presenting with markedly elevated values. The mean serum iron level was 52.1 µg/dL, with a mean total iron-binding capacity (TIBC) of 228.9 µg/dL and a mean ferritin level of 1174.6 µg/dL.

MCM was diagnosed by fungal culture and/or histopathology. Histopathologic evidence of tissue invasion by fungal elements was found in 93% of cases. Among those, 88% demonstrated necrosis and 37% showed angioinvasion. In three cases (7%), histopathologic confirmation was reported from outside facilities, but primary documentation was unavailable in the local medical record. These patients were included based on compatible clinical, radiologic, and operative findings, supported by either outside pathology reports, culture results, or intraoperative descriptions consistent with invasive mucormycosis. Fungal cultures were positive in 20 of 45 patients (44%). Among the *Mucorales* isolates, 45% were identified as *Rhizopus* species (unspecified), 25% as *Rhizopus arrhizus*, 25% as other (unspecified), and 5% as *Absidia* spp. (now *Lichtheimia* spp.).

Initial therapy in most cases included intravenous liposomal amphotericin B (Ambisome) at a dose of 5 mg/kg daily; one patient received 10 mg/kg, and another received a fixed dose of 600 mg IV daily. Nearly all patients also underwent surgical debridement, often requiring multiple procedures. The mean amphotericin B duration was 24 days (range: 1–90). Ninety-one percent of patients underwent surgery, with an average of three procedures per patient (range: 1–12). Amphotericin-related toxicity occurred in 16% of patients, primarily hypokalemia (42%) and acute kidney injury (42%).

EQUAL scores for Mucormycosis had a median of 19 (IQR, 16–22).

The in-hospital case fatality rate was 42%. However, when early post-discharge deaths were included, the overall mortality increased to 76%, leaving only six patients with documented short-term follow-up ranging from 7 days to 3 weeks after diagnosis. An additional five patients were discharged to hospice or lost to follow-up. Mortality was 100% among patients with DKA, compared to 31% in those without DKA (*p* = 0.001) (Table 2). Patients with histopathologic angioinvasion had significantly higher mortality (69% vs. 28%, *p* = 0.015), and those with ROCM had a mortality rate of 70% (*p* = 0.019). No statistically significant differences were observed between these iron parameters and clinical outcomes. Non-survivors had significantly higher median HbA1c values than survivors (9.5 vs. 6.4, *p* = 0.004).

This table outlines the clinical manifestations, diagnostic modalities, therapeutic interventions, and in-hospital mortality. Statistically significant differences in outcomes are presented for key risk factors including diabetic ketoacidosis (DKA), histopathologic angioinvasion, and mucormycosis subtype. *p*-values represent comparisons between survivors and non-survivors.

**Figure 3:**
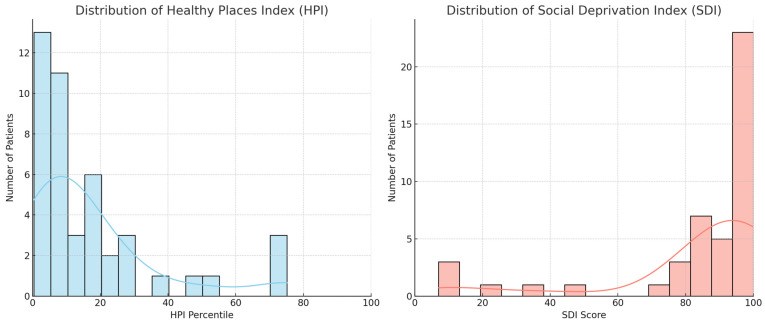
Distribution of Healthy Places Index (HPI) and Social Deprivation Index (SDI) Scores Among Patients with Mucormycosis.

Among the 45 patients in our cohort, residential ZIP code analysis revealed significant social disadvantage. Patients resided in 29 different ZIP codes spanning several counties across Central California. Based on available ZIP Code Tabulation Area (ZCTA) data: Healthy Places Index (HPI) scores ranged from 0.4 to 75.1, with a median of 10.05 and interquartile range (IQR) of 2.7–20.7 (Figure 3). Notably, 34 of 44 patients (1 missing data) (77%) with available HPI data resided in areas at or below the 25th HPI percentile, indicating neighborhoods with markedly low health opportunity. An HPI of 1.1, for example, reflects that the ZIP code has healthier community conditions than only 1.1% of other California ZIP codes. Social Deprivation Index (SDI) scores ranged from 7 to 100, with a median of 94.0 and IQR of 83.0–99.0. 29 of 45 patients (64%) lived in areas with SDI scores ≥ 85, reflecting elevated levels of social deprivation.

The figure depicts the distribution of neighborhood-level health equity indicators among 45 patients with mucormycosis treated at a referral center in Central California. HPI (left panel) is a percentile-based measure where lower scores indicate greater disadvantage and poorer community conditions. SDI (right panel) is a composite score ranging from 0 to 100, with higher scores indicating greater social deprivation. Each dot represents an individual patient’s residential ZIP Code Tabulation Area (ZCTA). Horizontal lines indicate the median and interquartile range.

## 4. Discussion

Mucormycosis (MCM) is an aggressive, angioinvasive fungal infection caused by molds of the order Mucorales, disproportionately affecting individuals with metabolic dysregulation or immunosuppression [19]. Established risk factors include uncontrolled DM, DKA, hematologic malignancies, organ transplantation, corticosteroid therapy, neutropenia, and trauma. Infection often begins in paranasal sinuses or lungs following inhalation of fungal spores, with rapid contiguous spread leading to vascular invasion and tissue necrosis [9]. Although invasive fungal infections are more commonly associated with neutropenic patients or transplant recipients in high-resource settings, extensive global data confirm poorly controlled diabetes mellitus as the most prevalent and potent risk factor for mucormycosis, particularly in low- and middle-income settings [4]. Mortality remains high across all forms, especially with disseminated disease, where rates exceed 90%.

While much of the published U.S. literature emphasizes MCM in the setting of hematologic malignancies or organ transplantation, several domestic studies have long recognized DM as a leading risk factor [20]. A Veterans Affairs cohort reported that 59% of MCM cases occurred in individuals with DM, surpassing leukemia (29%) [21]. Our cohort further diverges from the typical malignancy-dominated U.S. profile, describing 96% of patients with DM and 69% with DM as the sole predisposing condition. This pattern aligns more closely with trends from lower- and middle-income countries (LMICs) such as India and Iran, where diabetes consistently dominates MCM risk profiles. Indian series have reported DM in 54–76% of cases, particularly among rhino-orbito-cerebral presentations, while an Iranian 21-year study identified DM in 18.5% of patients, second only to hematologic malignancy [22,23].

Although only eight patients in our cohort had confirmed SARS-CoV-2 infection prior to MCM diagnosis, we observed a notable spike in cases during the first year of the COVID-19 pandemic. We hypothesize that this increase may reflect the indirect consequences of the pandemic, particularly disruptions in outpatient diabetes care, limited access to chronic disease management services, and delays in seeking medical attention due to fear of exposure. Such factors may have contributed to worsening glycemic control in already vulnerable populations, thereby increasing the risk for invasive fungal infections such as MCM. Similar trends have been observed in other global and U.S. cohorts, suggesting that the pandemic amplified preexisting structural vulnerabilities and metabolic risk factors, even in the absence of direct COVID-19 infection [24,25]. An earlier, smaller increase in cases was also noted in 2017. As this was a retrospective study, we were unable to determine a specific cause for that spike, although environmental or reporting factors may have contributed.

The pathogenesis of MCM is shaped by a triad of hyperglycemia, acidosis, and iron dysregulation [7,26]. DKA, in particular, creates a permissive environment for fungal proliferation: acidosis impairs neutrophil chemotaxis and oxidative burst, while ketone bodies upregulate fungal expression of high-affinity iron permeases (FTR1), enabling iron acquisition from host tissues [26,27]. Our findings support this mechanistic model: 19% of patients presented in DKA, and all experienced in-hospital mortality. This 100% mortality mirrors reports from Mexico and India, where DKA is repeatedly associated with fatal outcomes [27,28]. Although we collected serum iron, ferritin, and TIBC, no statistically significant association with mortality was found, likely due to the small sample size and the absence of labile plasma iron (LPI) measurements. Angioinvasion, histologically confirmed in 62% of cases, was associated with a 2.5-fold increase in mortality (69% vs. 28%, *p* = 0.015), reinforcing its role as a key virulence determinant [8]. The predominance of ROCM (60%) and its associated 70% mortality in our cohort reflect the lethal interplay between sinus-based disease, angioinvasion, and uncontrolled metabolic disease.

Glycemic control, as measured by hemoglobin A1c (HbA1c), is a critical modifiable determinant of MCM outcomes. In our cohort, 36% of diabetic patients had HbA1c > 10%, and over 60% had HbA1c > 8%, indicating widespread poor glycemic control despite access to a developed healthcare system. These levels far exceed the 7–8% threshold associated with impaired neutrophil function and increased susceptibility to invasive fungal infections. A national multicenter study from Mexico by Jiménez-Jacinto et al. (preprint) similarly found significantly higher mortality among patients with HbA1c > 9%, particularly when antifungal therapy was delayed [29]. Although conducted in Mexico, these findings are relevant to our Central California cohort, which shares a predominantly Hispanic population and faces comparable barriers to specialty care and chronic disease management. Our data provide corroborative U.S.-based evidence that suboptimal glycemic control is both prevalent and prognostically significant in MCM. While we could not assess access to outpatient diabetes care due to the retrospective nature of this study, the high HbA1c levels at presentation suggest missed opportunities for chronic disease intervention.

Over 60% of patients in our cohort identified as Hispanic, reflecting both the demographic composition of California’s Central Valley and the disproportionate burden of diabetes in this population. Hispanic communities face elevated risk for diabetes-related complications due to a complex interplay of genetic predisposition, environmental exposures, and socioeconomic barriers [30,31]. Hispanic adults experience higher rates of obesity, insulin resistance, and diabetes complications compared to non-Hispanic whites [32]. These metabolic risks are compounded by structural inequities, including limited insurance coverage, language barriers, low health literacy, and reduced access to specialty care such as endocrinology.

To further assess how place-based disadvantage may influence MCM risk and outcomes, we applied two validated ZIP code–level indices: the Social Deprivation Index (SDI) and the California Healthy Places Index (HPI). Over 75% of patients lived in neighborhoods with HPI scores below the 25th percentile, and 64% resided in SDI ≥ 85 zones, indicating widespread barriers in housing, education, transportation, and healthcare access. These data mirror broader characterizations of the Central Valley as a “patchwork of poverty and prosperity,” where rural, agricultural communities suffer entrenched disparities in chronic disease outcomes [12]. In this context, MCM emerges not only as an opportunistic fungal infection, but as a sentinel of systemic failures in chronic disease prevention and healthcare equity.

Management of mucormycosis requires rapid initiation of active antifungal therapy, surgical debridement of necrotic tissue, and reversal of underlying predisposing factors such as diabetic ketoacidosis or immunosuppression [33]. In our cohort, nearly all patients received amphotericin B as initial therapy, with a mean duration of 24 days, and 91% underwent surgical debridement, often requiring multiple procedures. Despite these measures, in-hospital mortality remained high (42%), underscoring the challenge of managing this infection even with timely and appropriate therapy.

To further assess the quality of diagnostic and therapeutic practices in our cohort, we applied the EQUAL Score for Mucormycosis. The median score was 19 (IQR, 16–22), comparable to values reported in real-world studies outside specialized mycology centers, where median scores typically range from 15 to 18 [34]. The maximum attainable score varies from 25 to 32 points, depending on diagnostic completeness and the availability of culture or histopathologic confirmation [15]. The EQUAL Mucormycosis Score was originally developed and validated in tertiary hematology and transplant centers participating in the ECMM Excellence network, where patients commonly experience prolonged neutropenia, receive mould-active prophylaxis, and have access to advanced diagnostics and serial imaging [34]. In contrast, our cohort consisted predominantly of patients with diabetes mellitus and rhino-orbito-cerebral mucormycosis treated across mixed-resource regional hospitals serving rural communities. In this context, several hematology-specific score items, such as prophylaxis for neutropenia, were not applicable, and diagnostic infrastructure (e.g., fungal PCR, susceptibility testing) was variably available. These contextual differences likely account for modestly lower total scores despite strong adherence to the key management principles of mucormycosis, including early surgical intervention, amphotericin-based therapy, and control of underlying metabolic risk factors. Applying the EQUAL framework in this real-world, nonhematologic population nonetheless provided valuable insight into diagnostic and therapeutic performance in a region where diabetes is the dominant risk factor.

This study has several important limitations. Its retrospective design limits causal inference and introduces potential selection and misclassification biases. Case identification relied on ICD coding and microbiology records, which may miss cases or overestimate burden, particularly given the absence of advanced diagnostic modalities such as PCR, MALDI-TOF, or next-generation sequencing. Culture-based confirmation may have missed histology-only cases, and labile plasma iron, a more relevant predictor of iron-related risk, was not available. Outcomes were limited to in-hospital mortality without long-term follow-up on relapses, visual loss, or facial disfigurement. Potential environmental exposures (e.g., agricultural work, dust storms, or flooding) were not captured. Finally, although we used ZIP code–level SDI and HPI as surrogate measures of SDOH, these cannot substitute for individual-level data on income, health literacy, or healthcare access, and may underrepresent intra-community variation.

Despite these limitations, our findings highlight the urgent need for multifaceted interventions in high-risk, underserved regions such as California’s Central Valley. Future prospective studies should incorporate serial HbA1c measurements, fungal burden quantification, antifungal pharmacokinetics, and iron metabolism panels. Research should also include more granular SDOH data and environmental exposure histories to better characterize at-risk populations. Community-based solutions, including mobile health clinics, culturally tailored diabetes education, and structured discharge planning with follow-up, may bridge current gaps in chronic disease management. To reduce MCM morbidity and mortality, public health approaches must extend beyond hospital walls and address the systemic and geographic inequities that shape infectious disease risk.

## 5. Conclusions

MCM in Central California represents not only a clinical challenge, but also a broader public health obstacle to effective chronic disease prevention and health equity. Our findings illustrate how poorly controlled diabetes, social deprivation, and limited access to care converge to drive severe fungal infections. Addressing MCM requires a dual focus on timely antifungal management and coordinated upstream interventions that target chronic disease disparities.

## Figures and Tables

**Table 1:** Demographics and Underlying Risk Factors Among Patients with Mucormycosis (N = 45).

Characteristics	N (%) or Median [IQR] £
Age (years)	53 [18–81]
Male	29 (64%)
Hispanic	28 (62%)
Diabetes Mellitus (DM)	43 (96%)
DM * only (no other risk factors)	31 (69%)
DM and other risk factors	12 (27%)
Diabetic ketoacidosis	8 (19%)
HbA1c > 7.5%	24 (58%)
HbA1c > 10%	15 (36%)
Mean HbA1c	9.5%
Median HbA1c (IQR)	7.8% (IQR, 6.7–12.6)
COVID±-19 history	8 (18%)
SDI Ɨ ≥ 85	29 (64%)
HPI ¶ < 25th percentile	34 (77%)

* Diabetes mellitus ± Coronavirus Disease Ɨ Social Deprivation Index. ¶ Healthy Places Index £ Inter Quartile Range.

**Table 2:** Clinical Presentation, Histopathologic Features, and Outcomes in Patients With Mucormycosis (N = 45).

Characteristic	N (%)	*p*-Value
Rhino-orbital-cerebral (ROCM)	27 (60%)	
Pulmonary	10 (22%)	—
Muscular	4 (9%)	—
Cutaneous	3 (7%)	—
Gastrointestinal	1 (2%)	—
Histopathologic invasion	42 (93%)	—
Necrosis	40 (88%)	—
Angioinvasion	17 (37%)	0.015
Surgery performed	41 (91%)	—
Mean number of surgeries	3	—
Amphotericin B given	43 (96%)	—
Amphotericin toxicity	7 (16%)	—
In-hospital mortality	19 (42%)	—
Mortality in DKA	100%	0.001
Mortality in ROCM	70%	0.019
Mortality with angioinvasion	69%	0.015

## Data Availability

The original contributions presented in this study are included in the article. Further inquiries can be directed to the corresponding authors.
